# 

**Published:** 1948

**Authors:** 


PUBLISHED UNDER THE AUSPICES OF THE BRISTOL MEDICO-CHIRURGICAL SOCIETY

### THE BRISTOL MEDICO-CHIRURGICAL JOURNAL

*A Journal of the Medical Sciences for the*

### WEST OF ENGLAND AND SOUTH WALES

*Editor:*
E. WATSON-WILLIAMS, *M.C.*, M.D., Ch.M., F.R.C.S.

*with whom are associated*

G. E. F. SUTTON, *M.C.*, M.D., F.R.C.P.

W. MELVILLE CAPPER, M.B., B.S., F.R.C.S.

N. S. CRAIG, *M.C.*, M.D., *Assistant Editor.*

*Local Correspondents:*

BATH—A. D. BATEMAN, *O.B.E.*, F.R.C.S.

CARDIFF—J. D. SPILLANE, M.D., M.R.C.P.

BRISTOL:
J. W. ARROWSMITH LTD., QUAY STREET AND SMALL STREET

Vol. LXV. No. 235. *Price:* FOUR SHILLINGS net. AUTUMN · 1948

